# Hfq regulates the expression of the thermostable direct hemolysin gene in *Vibrio parahaemolyticus*

**DOI:** 10.1186/1471-2180-8-155

**Published:** 2008-09-21

**Authors:** Masayuki Nakano, Akira Takahashi, Zehong Su, Nagakatsu Harada, Kazuaki Mawatari, Yutaka Nakaya

**Affiliations:** 1 Department of Nutrition and Metabolism, Institute of Health Biosciences, The University of Tokushima Graduate School, Tokushima, Japan

## Abstract

**Background:**

The *hfq *gene is conserved in a wide variety of bacteria and Hfq is involved in many cellular functions such as stress responses and the regulation of gene expression. It has also been reported that Hfq is involved in bacterial pathogenicity. However, it is not clear whether Hfq regulates virulence in *Vibrio parahaemolyticus*. To evaluate this, we investigated the effect of Hfq on the expression of virulence-associated genes including thermostable direct hemolysin (TDH), which is considered to be an important virulence factor in *V. parahaemolyticus*, using an *hfq *deletion mutant.

**Results:**

The production of TDH in the *hfq *deletion mutant was much higher than in the parental strain. Quantification of *tdh *promoter activity and mRNA demonstrated that transcription of the *tdh *gene was up-regulated in the mutant strain. The *hfq*-complemented strain had a normal (parental) amount of *tdh *expression. The transcriptional activity of *tdhA *was particularly increased in the mutant strain. These results indicate that Hfq is closely associated with the expression level of the *tdh *gene. Interestingly, other genes involved in the pathogenicity of *V. parahaemolyticus*, such as VP1680, *vopC*, and *vopT*, were also up-regulated in the mutant strain.

**Conclusion:**

Hfq regulates the expression of virulence-associated factors such as TDH and may be involved in the pathogenicity of *V. parahaemolyticus*.

## Background

*Vibrio parahaemolyticus *is a major causative agent of gastroenteritis, and is often associated with the consumption of raw or undercooked shellfish. In addition to gastroenteritis, this organism causes clinical manifestations such as wound infections and septicemia [1-3]. Most clinical isolates of *V. parahaemolyticus *exhibit β-type hemolysis on a special blood agar medium, whereas environmental isolates do not. This hemolysis is called the Kanagawa phenomenon (KP) and is considered to be a reliable marker for detection and identification of pathogenic strains [4]. It has been reported that thermostable direct hemolysin (TDH) specifically contributes to the KP [2]. Previous studies have shown that TDH shows several biological activities such as hemolysis, cell cytotoxicity and enterotoxicity [5-7]. Furthermore, molecular epidemiological studies have indicated that the *tdh *gene is strongly associated with clinical strains [8]. Therefore, TDH is thought to be an important virulence factor in *V. parahaemolyticus*. Although the genetic structures of the *tdh *gene and its neighboring regions are known, there is little information as to how *tdh *gene expression is controlled by other factors in this organism.

The *hfq *gene is conserved in a wide range of bacteria, but Hfq varies in the number of amino acids between different bacteria [9]. Hfq is a global regulator that modulates the stability and translation of mRNAs. It is thought that Hfq acts as an RNA chaperone by mediating interactions between many small regulatory RNAs and their mRNA targets [9]. It has been shown that Hfq regulates the expression of many genes, including the genes encoding for stationary-phase sigma factor (*rpoS*) and outer membrane protein A (*ompA*) [10-12]. It has also been shown that Hfq contributes to pathogenicity in bacteria such as *Brucella abortus, Listeria monocytogenes*, *Pseudomonas aeruginosa, Salmonella typhimurium*, *V. cholerae*, and *Yersinia enterocolitica *[13-18]. Although Hfq is associated with bacterial virulence, it is not clear whether Hfq is associated with pathogenicity in *V. parahaemolyticus*.

In this study, we have attempted to elucidate the correlation between Hfq and *V. parahaemolyticus *pathogenicity, particularly with regard to the important virulence factor TDH, using an *hfq *gene-deletion mutant.

## Results

### Effect of Hfq on growth of *V. parahaemolyticus*

**Figure 1:**
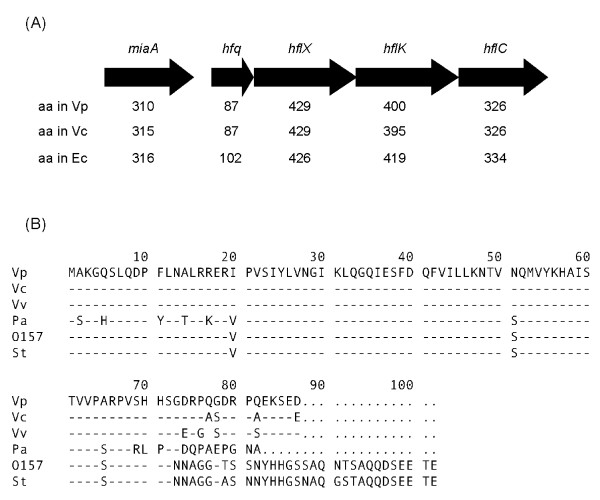
**Genetic structure and sequence alignment of Hfq**. (A) Genetic structure of *hfq *and neighboring regions. Vp, *V. parahaemolyticus *strain RIMD2210633 (accession no. BA000032); Vc, *V. cholerae *(accession no. NP_230001); Ec, *E. coli *serotype O157:H7 (accession no. NP_313175). Arrows indicate the direction of transcription. The numbers of amino acids (aa) that comprise the proteins are indicated. (B) Sequence alignment of Hfq from various pathogens. Dashes indicate amino acid residues that are identical to those in *V. parahaemolyticus*. All Hfq amino acid sequences were obtained from GenBank. Vp, *V. parahaemolyticus *strain RIMD2210633 (accession no. BA000032); Vc, *V. cholerae *(accession no. NP_230001); Vv, *V. vulnificus *(accession no. NP_760222); Pa, *P. aeruginosa *(accession no. NP_233631); O157, *E. coli *serotype O157:H7 (accession no. NP_313175); St, *S. typhi *(accession no. NP_458797).

The complete genome of *V. parahaemolyticus *strain RIMD2210633 has been sequenced, and the *hfq *gene is located on chromosome 1 (GenBank: BA000031) [19]. The gene structures neighboring *hfq *in *V. parahaemolyticus *are identical to those of *V. cholerae *and *Escherichia coli *(Fig. 1A) [14], and the Hfq amino acid sequence of *V. parahaemolyticus *is highly conserved with that of *V. cholerae *(Fig. 1B).

**Figure 2:**
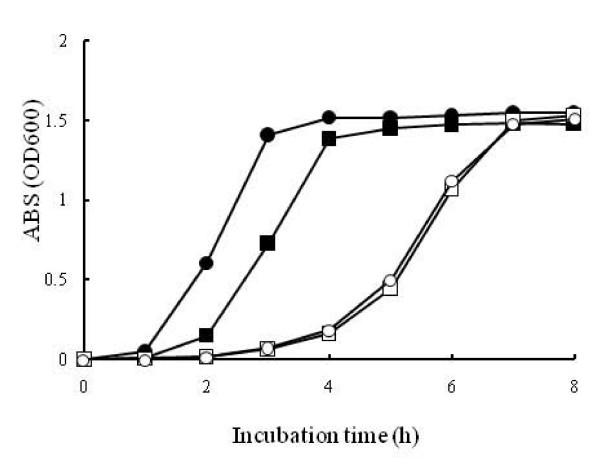
**Growth of the Δ*hfq *strain in LB medium**. Bacteria were cultured in LB medium supplemented with 3% NaCl at 37°C and the cell density was measured with a spectrophotometer. The assay was performed independently 3 times. Closed circle, parental strain; closed square, *hfq*-complemented deletion strain (pHfqΔ*hfq*); open circle, *hfq *deletion strain (Δ*hfq*); open square, *hfq *deletion strain with empty vector (pSN1Δ*hfq*).

Previous studies showed that defects in the *hfq *gene decreased the growth rates of *E. coli *and *P. aeruginosa*, but not of *S. typhimurium *and *V. cholerae *[11,14,15,20]. In LB medium supplemented with 3% NaCl, Δ*hfq*, a derivative of the *V. parahaemolyticus *strain RIMD2210633 with an in-frame deletion of 85 of the 87 amino acid residues of Hfq, showed a reduced growth rate as compared with that of the parental strain (Fig. 2). This indicates that Hfq has an effect on the growth of *V. parahaemolyticus*.

### Hemolytic activity of secreted protein from Δhfq

**Figure 3:**
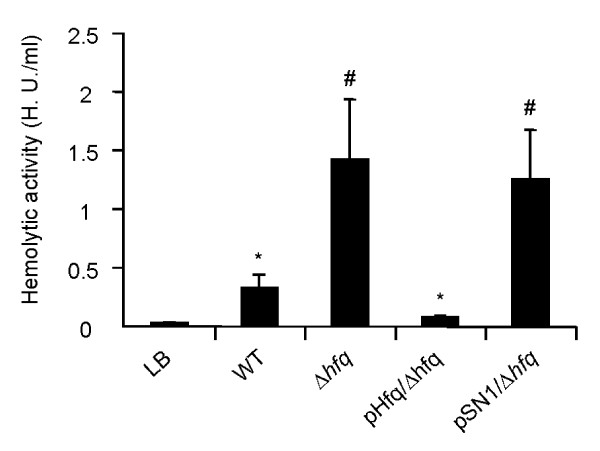
**Hemolytic activity in culture supernatants of parental and derivative strains**. Bacteria were cultured in 10 ml of LB medium supplemented with 3% NaCl at 37°C. One hemolytic unit (HU) was defined as in a previous report [45,46]. The results are expressed as means ± SDs of 5 independent experiments. *, p < 0.05 versus LB; #, p < 0.01 versus WT. LB, negative control; WT, *V. parahaemolyticus *strain RIMD2210633 (parent strain); Δ*hfq*, *hfq *deletion strain; pHfq/Δ*hfq*, *hfq*-complemented deletion strain; pSN1/Δ*hfq*, deletion strain with empty vector.

Hfq regulates the bacterial pathogenicity that is mediated by alterations in virulence gene expression [13-18]. Therefore, we investigated the hemolytic activity of the Δ*hfq *strain by determining the level of TDH in culture supernatants, because TDH is thought to be important for *V. parahaemolyticus *pathogenicity. In the following experiments, we evaluated all of the strains under the same growth phase conditions. The relative hemolytic activity in the culture supernatant of the Δ*hfq *strain was significantly higher than that of the parental strain. We introduced pHfq, which harbors the *hfq *gene, into Δ*hfq *and showed that the relative hemolytic activity in the culture supernatant of pHfq/Δ*hfq *was lower than that of Δ*hfq*. The hemolytic activity in the culture supernatant of pSN1/Δ*hfq*, in which an empty vector was introduced into Δ*hfq*, was almost the same as that of Δ*hfq *(Fig. 3). These results suggest that Hfq is associated with the production of TDH in *V. parahaemolyticus*.

### Hfq is involved in the expression of TDH

**Figure 4:**
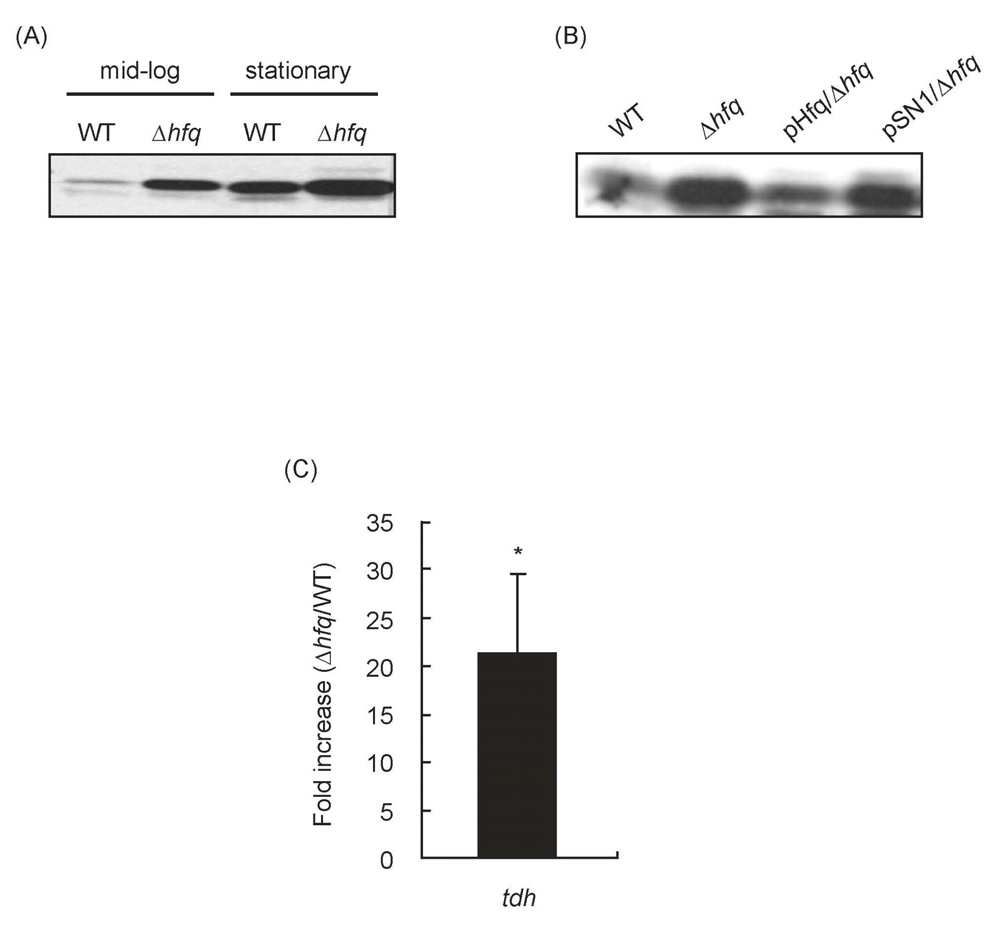
**Production of TDH by the Δ*hfq *strain**. (A) Detection of TDH in cell lysates from mid-log phase (OD_600 _= 0.6) and stationary phase (OD_600 _= 1.5) cultures. (B) Detection of TDH in mid-log phase cell lysates from the parent strain and derivatives. (C) Quantification of *tdh *mRNA. Fold change in the gene transcription level from mid-log phase in the Δ*hfq *strain versus the parent strain, as measured by quantitative real-time RT-PCR. Data analysis was as previously described [42,43]. Data are expressed as means ± SDs of 5 independent experiments. *, *p *< 0.05.

We next investigated the level of TDH by Western blotting, and found that the expression of TDH was increased at mid-log growth phase (OD_600 _= 0.6) as compared with the parental strain (Fig. 4A). The amount of TDH in the Δ*hfq *strain at stationary growth phase was also higher than that of the parent strain (Fig. 4A). The elevation in TDH production was fully reversed by trans-complementation with the *hfq *gene in pHfq/Δ*hfq *(Fig. 4B). These results suggest that TDH production is specifically associated with Hfq. We also examined the mRNA levels of the *tdh *gene at mid-log growth phase using quantitative real-time RT-PCR. The amount of *tdh *mRNA in the Δ*hfq *strain was significantly higher than in the parent strain (Fig. 4C). These results suggest that the elevation of hemolytic activity in the Δ*hfq *strain is due to activation of *tdh *gene transcription.

### Transcriptional levels of the tdh genes in Δhfq

**Figure 5:**
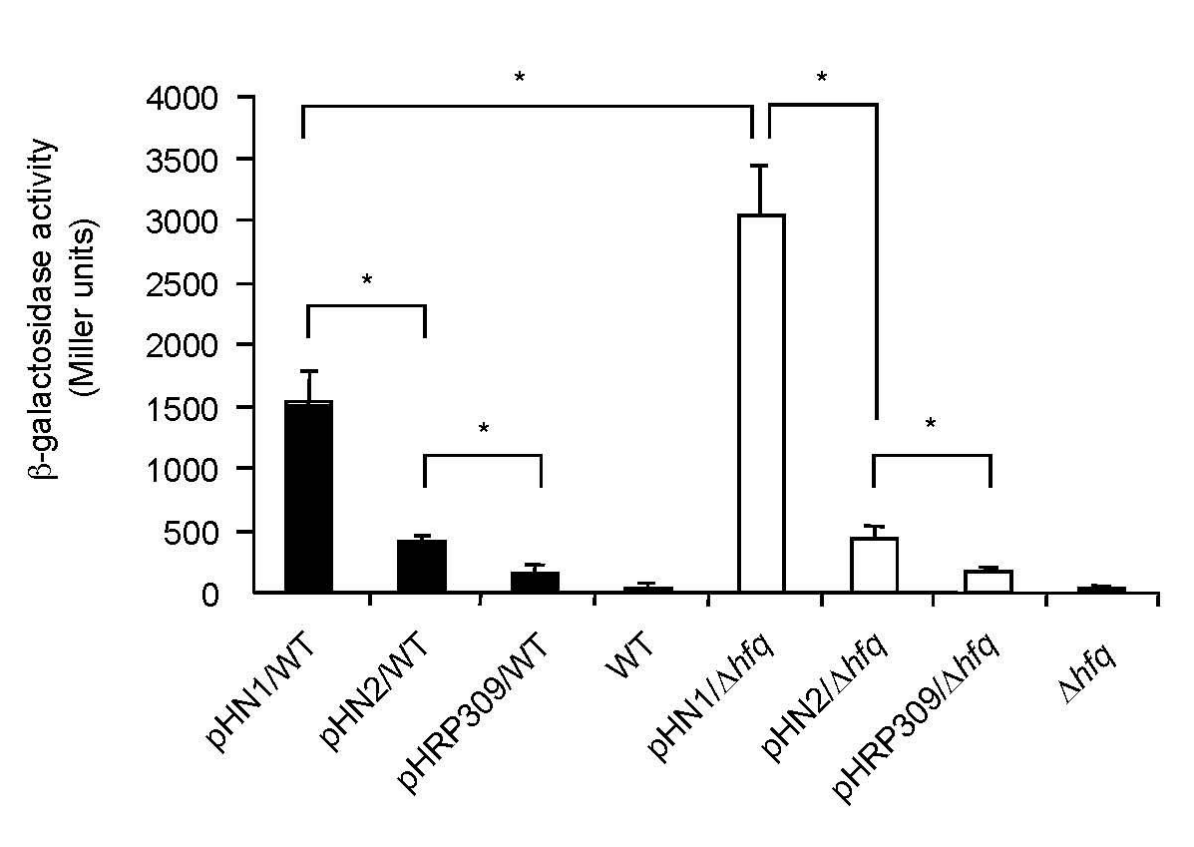
**Activities of the *tdhA *and *tdhS *gene promoters in the WT and Δ*hfq *strains**. β-galactosidase activity measured in the parental WT strain (black bars) and the Δ*hfq *strain (open bars). pHN1/WT, pHN1 harboring *tdhA *gene promoter in *V. parahaemolyticus *strain RIMD2210633; pHN2/WT, pHN2 harboring *tdhS *gene promoter in *V. parahaemolyticus *strain RIMD2210633; pHRP309/WT, pHRP309 in *V. parahaemolyticus *strain RIMD2210633; pHN1/Δ*hfq*, pHN1 harboring *tdhA *gene promoter in Δ*hfq *strain; pHN2/Δ*hfq*, pHN2 harboring *tdhS *gene promoter in Δ*hfq *strain; pHRP309/Δ*hfq*, pHRP309 in Δ*hfq *strain. Data in Miller units are the means ± SDs of 5 independent experiments. *, *P *<0.01.

Previous studies showed that while two *tdh *genes (*tdhA *and *tdhS*) are located on chromosome 2 of strain RIMD2210633, in this strain TDH production was attributed only to *tdhA *[15,18]. Although we found that TDH was elevated significantly at both the transcriptional and translational levels in the Δ*hfq *strain, quantitative real-time RT-PCR and Western blotting using anti-TDH antibody cannot distinguish between the two *tdh *genes, since their nucleotide sequences are very similar (97% identity). To solve this problem, transcriptional fusions of *tdhA *and *tdhS *to the *lacZ *reporter gene (pHN1 and pHN2, respectively; Table 1) were constructed and were monitored in Δ*hfq *or in the isogenic parental strains. In the parental strain, the relative activity of the *tdhA*-*lacZ *fusion gene was much higher than the *tdhS-lacZ *fusion gene (Fig. 5). This result is consistent with previous reports [21,22]. Interestingly, the relative activity of the *tdhA-lacZ *fusion gene in the Δ*hfq *strain was significantly elevated when compared with the parental strain, whereas there was no difference in the activity of the *tdhS-lacZ *fusion gene between the parental and Δ*hfq *strains (Fig. 5). These results indicate that the elevation of *tdh *mRNA levels in the Δ*hfq *strain is due to *tdhA *gene transcription.

**Table 1:** Bacteria and plasmids

Bacteria and plasmids	Comments^a^	References
*V. parahaemolyticus*		
RIMD2210633	*tdh*^+ ^(serotype O3:K6; clinical isolate)	19
Δ*hfq*	*hfq *mutant derived from RIMD2210633	This study
pHfq/Δ*hfq*	Δ*hfq *harboring pHfq	This study
pSN1/Δ*hfq*	Δ*hfq *harboring pSN1	This study
pHN1/WT	RIMD2210633 harboring pHN1	This study
pHN2/WT	RIMD2210633 harboring pHN2	This study
pHRP309/WT	RIMD2210633 harboring pHRP309	This study
pHN1/Δ*hfq*	Δ*hfq *harboring pHN1	This study
pHN2/Δ*hfq*	Δ*hfq *harboring pHN2	This study
pHRP309/Δ*hfq*	Δ*hfq *harboring pRP309	This study
*E. coli*		
SM10λ*pir*	R6K*ori *λ*pir supE thi thr leu lacY supE*	39
Plasmids		
pYK1	Suicide vector; Cm^r^	39
pSA19CP-MCS	For gene complementation; Cm^r^	40
pHRP309	For β-galactosidase assay; *lacZ gen*	47
pSN1	pSA19CP-MCS containing PCR-generated *tdhA *promoter	23
pHfq	pSN1 containing PCR-generated *hfq*	This study
pHN1	pHRP309 containing PCR-generated *tdhA *promoter	This study
pHN2	pHRP309 containing PCR-generated *tdhS *promoter	This study

^a^Cm^r^, chloramphenicol resistance

### Hfq regulates the expression of other virulence-associated genes

**Figure 6:**
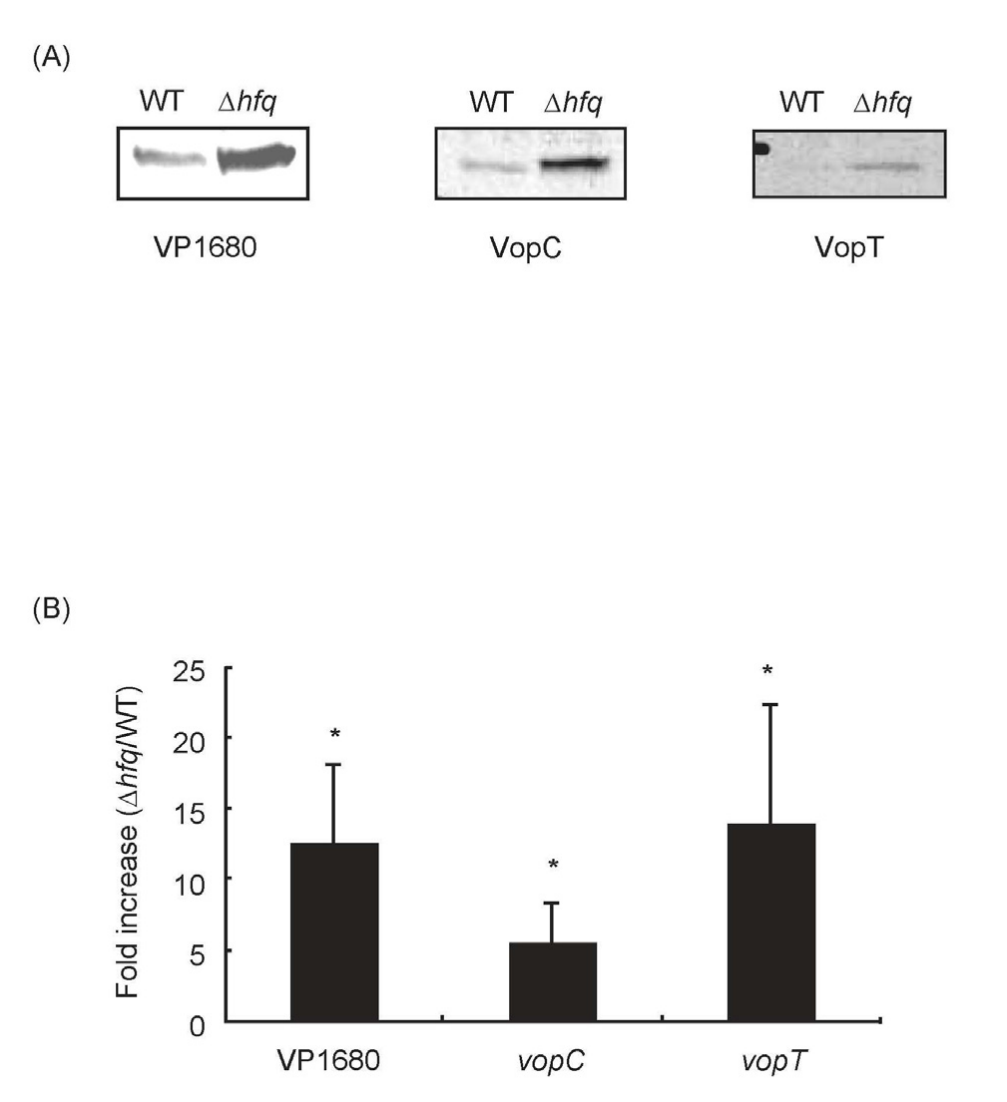
**Expression of virulence-associated genes in the Δ*hfq *strain**. (A) Western blot detection of proteins in total cell lysates. (B) Quantification of mRNAs encoding virulence-associated proteins. The fold changes in gene transcription levels in the Δ*hfq *and parental WT strains grown to mid-log phase were determined by quantitative real-time RT-PCR. Data analysis was as previously described [42,43]. Data are the means ± SDs of 5 independent experiments. *, *p *< 0.05.

Recently, it has been shown that type III secretion systems (TTSS1 and TTSS2) in *V. parahaemolyticus *are also important for the pathogenicity of this organism [23]. To evaluate whether Hfq can regulate the expression of TTSS genes as well as *tdh*, we investigated the expression of three genes (VP1680, *vopC*, and *vopT*), which encode TTSS effector proteins [24,25], at the transcriptional and translational levels in the Δ*hfq *strain. VP1680, which is secreted via TTSS1, showed elevated gene expression in the Δ*hfq *strain. The expression of *vopC *and *vopT*, which are secreted via TTSS2, was also increased in the Δ*hfq *strain (Fig. 6). These results indicate that the transcription of some genes encoding TTSS effector proteins is regulated in an Hfq-dependent manner.

## Discussion

Hfq plays a role as a major post-transcriptional regulator in numerous bacterial pathways [9]. Previous studies have shown that Hfq affects the virulence of various pathogenic bacteria [13-18]. In this study, we have demonstrated that Hfq plays a critical role in the expression of TDH (Fig. 4). This indicates that Hfq is involved in the virulence of *V. parahaemolyticus *just as in other pathogenic bacteria, because TDH is considered to be an important virulence factor in *V. parahaemolyticus *[5-8]. The modulation of bacterial pathogenicity by Hfq has been associated with small non-coding RNAs [26-29]. It has been shown that deletion of the *hfq *gene in *P. aeruginosa *affects the expression of virulence genes by changing the stability of a small non-coding RNA [26]. Recently, Lenz *et al*. have speculated that small non-coding RNAs exist in the *V. parahaemolyticus *genome [29]. Although there is little information regarding small non-coding RNAs in *V. parahaemolyticus*, we speculate that one or more small non-coding RNAs interacting with *tdh *may regulate virulence gene expression levels.

The production of TDH is reportedly affected by environmental factors such as pH, temperature, and chemicals [30-34], but little is known about the modulation of expression of the *tdh *gene by internal factors in *V. parahaemolyticus*. In this study, we showed that Hfq in *V. parahaemolyticus *was important for the expression of the *tdh *gene and that the transcription of the *tdhA *gene rather than the *tdhS *gene was regulated by Hfq (Figs. 4 and 5). This result indicates that the expression of the *tdh *gene is under the control of Hfq. Lin *et al*. have reported that ToxR in *V. parahaemolyticu*s is associated with the production of TDH [35]. We investigated the transcriptional activity of *toxR *in the Δ*hfq *strain under our experimental conditions, but could not find any differences between the Δ*hfq *and wild type strains (data not shown). This suggests that the regulation of *tdh *gene expression by Hfq is *toxR*-independent. We are currently examining how Hfq regulates the expression of *tdh*.

Proteome and microarray analyses in various bacteria have shown that Hfq controls the expression of various genes [14,16,26]. Thus, Hfq is considered to be a global regulator of gene expression including genes encoding virulence factors. In this study, we confirmed that Hfq also regulates the expression of at least three effector proteins of TTSSs, namely VP1680, VopC and VopT, in *V. parahaemolyticus *(Fig. 6). It has been shown that TTSSs and these three effector proteins contribute to pathogenicity in *V. parahaemolyticus *[23-25]. Although TTSSs in pathogenic *E. coli *and *Salmonella *are involved in the regulatory mechanisms for the expression of TTSS-related genes [36,37], this is not yet clear in *V. parahaemolyticus*. Moreover, it seems likely that other novel effector proteins will be found that contribute to the pathogenicity of *V. parahaemolyticus *[25,38]. Our results suggest that Hfq may be important for the pathogenicity of *V. parahaemolyticus*. Experiments are currently underway to analyze the mechanisms by which Hfq governs the expression of virulence genes in *V. parahaemolyticus*.

## Conclusion

We have confirmed that the expression of TDH in *V. parahaemolyticus *is closely associated with the transcriptional activity of *tdhA*. Hfq regulates the transcriptional activity of *tdhA *and the expression of TDH in *V. parahaemolyticus*. We predict that Hfq may be a global regulator of pathogenicity in *V. parahaemolyticus*.

## Methods

### Bacteria and plasmids

The bacteria and plasmids used in this study are listed in Table 1. *V. parahaemolyticus *strain RIMD2210633 was used as the standard strain in this study [19]. *E. coli *and *V. parahaemolyticus *strains were routinely cultured in Luria-Bertani (LB) medium and LB medium supplemented with 3% NaCl, respectively.

### Construction of the *hfq *deletion mutant

The mutant strain was constructed by homologous recombination [23,39]. In brief, a DNA fragment was generated by PCR using primers hfq-1 (5'-GGATCCAGCCGATAACATTGAGCAGGC-3') and hfq-4 (5'-CTGCAGTCAGCACCAGTCAAATGTAC-3') after the preparation of PCR products using primers hfq-1 and hfq-2 (5'-CTCGATGAAGAATTAAGCCATTTTATTTTC-3'), and hfq-3 (5'-GAAAATAAAATGGCTTAATTCTTCATCGAG-3') and hfq-4. The amplified PCR product was cloned into the suicide vector pYAK1 [39] and the resulting plasmid was transformed into *E. coli *strain SM10 λ*pir*. Bacterial conjugation was performed with the recombinant *E coli *strain SM10 λ*pir *and *V. parahaemolyticus*. Colonies were confirmed to have the gene deletion by PCR using the primers hfq-1 and hfq-4 and by Southern blotting.

### Complementation of the *hfq *deletion mutant

Bacterial strains harboring the *hfq *gene were constructed as previously described [23]. Since the *hfq *gene promoter of *V. parahaemolyticus *has not been identified, the *tdhA *gene promoter was used for expressing *hfq *[23]. The *hfq *gene was amplified by PCR using primers hfq-5 (5'-GGATCCATGGCTAAGGGGCAATCT-3') and hfq-6 (5'-GAATTCTTAATCTTCTGATTTCTC-3'), and the PCR-generated *hfq *gene and *tdhA *promoter were cloned into pSA19CP-MCS to construct pHfq [40]. The constructed plasmid was introduced into the *V. parahaemolyticus *deletion mutant by electroporation [41].

### Quantitative real-time RT-PCR

Preparation of total RNA from *V. parahaemolyticus *was carried out previously [42]. Quantitative real-time RT-PCR and data analysis were performed as previously described [42,43]. The oligonucleotide primers for *rrs*, *tdh*, *vopC *(= *vpa1321*), and *vopT *(= *vpa1327*) were as previously described [42]. The oligonucleotide primers for VP1680 were as follows: 5'-CACACTTGGTATGGCGACAG-3' and 5'-TGAGTTGGTCTTGCACTTCG-3'. The oligonucleotide primers were designed using the genome sequence of *V. parahaemolyticus *strain RIMD2210633 [19].

### Preparation of polyclonal antibodies

Antibody against TDH was prepared as described previously [44]. Antibodies against VP1680, VopC, and VopT were prepared according to a previous report [23]. In brief, DNAs encoding VP1680, *vopC*, and *vopT *were generated by PCR using the following oligonucleotide primers: for VP1680, 5'-GGATCCATGGTGAATACAACGCAAAA-3' and 5'-GAATTCTTAAATCCAGCCTTCGGCT-3'; for *vopC*, 5'-GGATCCATGCCAATATTAAATATTAG-3' and 5'-GAATTCTTAACCATTTTTCAATAGCA-3'; for *vopT*, 5'-GGATCCGTGAAGGTTTGTAGAATACA-3' and 5'-GAATTCTCACTTAGCTAAATCTAGCG-3'. The resulting DNAs were cloned into pET30a. Recombinant proteins were expressed in *E. coli *BL21(DE3) and purified using His-tag affinity resins and buffer kits according to the manufacturers' instructions (Merck, Darmstadt, German). Polyclonal antibodies were generated by immunizing female New Zealand White rabbits as previously described [23].

### Western blotting

*V. parahaemolyticus *was cultured in LB medium supplemented with 3% NaCl. Cells (2 ml of culture) were collected by centrifugation and washed once with phosphate-buffered saline (PBS; pH 7.4). The pellets were resuspended in PBS and 10 μg of total cell lysates were used for Western blotting. After electrophoresis, proteins were transferred to membranes and probed with each of the primary antibodies. The secondary antibody was anti-rabbit IgG conjugated to horseradish peroxidase (Bioscience, Camarillo, CA, USA). Signals were detected using an ECL Western blotting kit according to the manufacturer's instructions (GE Healthcare Bio-Science, Piscataway, NJ, USA).

### Determination of hemolytic activity

Bacterial cells were prepared by diluting overnight cultures 1:1000 into fresh LB medium supplemented with 3% NaCl and incubating at 37°C for 24 h with shaking. After the elimination of bacterial cells from culture supernatants by centrifugation and a syringe filter, the supernatants were concentrated using an Amicon Ultra-15 centrifugal filter (Millipore, Billerica, MA, USA). Protein concentrations were determined using a BCA protein assay kit (Thermo Scientific, Rockford, IL, USA).

Hemolytic activity was assayed according to a previously described method [45,46] with rabbit red blood cells (rRBC) (Nippon Bio-Test Laboratories, Tokyo, Japan). Protein (20 μg) was incubated with rRBC at 37°C for 6 h.

### β-galactosidase assay

β-galactosidase assays were performed as described previously [47]. In brief, the *tdhA *and *tdhS *promoter regions were generated by PCR using the oligonucleotide primers tdhA-1 (5'-GTCGACAATTCACGACGAATCGGAG-3') and tdhA-2 (5'-GAATTCATATCGGTACTTCATAAA-3'), and tdhS-1 (5'-GTCGACAGCTTACAGCTTGGTATGCC-3') and tdhS-2 (5'-GAATTCGTGTTTCATAAAAAACCCAT-3'), respectively. The products were cloned into pHRP309 [48]. The constructed plasmids were introduced into *V. parahaemolyticus *strains by electroporation [41]. For this assay, bacteria were cultured in LB medium supplemented with 3% NaCl until early-log phase (OD_600 _= 0.2). The β-galactosidase activities of the strains were determined as described previously [49].

### Data analysis

All data are expressed as means ± standard deviations. Statistical significance was calculated by paired and unpaired *t*-tests. In all cases, a *p*-value of < 0.05 was considered significant.

## Authors' contributions

MN, AT, and ZS performed the experiments and drafted the manuscript. NH and KM participated in the discussions of the study design and provided technical support. YN conceived of the study and drafted the manuscript. All authors have read and approved the final manuscript.
